# To bloom once or more times: the reblooming mechanisms of *Iris germanica* revealed by transcriptome profiling

**DOI:** 10.1186/s12864-020-06951-x

**Published:** 2020-08-12

**Authors:** Zhuping Fan, Yike Gao, Yi Ren, Chunjing Guan, Rong Liu, Qixiang Zhang

**Affiliations:** Beijing Key Laboratory of Ornamental Plants Germplasm Innovation & Molecular Breeding, National Engineering Research Center for Floriculture, School of Landscape Architecture, Beijing Forestry University, No. 35 Qinghua East Road, Haidian District, Beijing, China

**Keywords:** *Iris germanica*, Reblooming, Transcriptome profiling, Floral initiation

## Abstract

**Background:**

The reblooming bearded iris (*Iris germanica*) can bloom twice a year, in spring and autumn. The extended ornamental period makes it more popular and brings additional commercial values. However, little is known about the reblooming mechanisms, making the breeding programs time-consuming and labor-wasting. Therefore, a comparative transcriptome profiling was conducted on once-bloomers and rebloomers from the same F_1_ generation on six development stages, and the candidate genes associated with reblooming were identified.

**Results:**

A total of 100,391 unigenes were generated, the mean length being 785 bp. In the three comparisons (the floral initiation stage of spring flowering in once-bloomers (OB-T1) vs the floral initiation stage of spring flowering in rebloomers (RB-T1); RB-T1 vs the floral initiation stage of autumn flowering in rebloomers (RB-T5); OB-T1 vs RB-T5), a total of 690, 3515 and 2941 differentially expressed genes (DEGs) were annotated against the public databases, respectively. The Gene Ontology (GO) and Kyoto Encyclopedia of Genes and Genomes (KEGG) analysis focused on the photoperiod response, the temperature insensitivity and the growth speed, to remove the redundant DEGs and figure out the candidate key genes. As a result, the following four genes, *PHYTOCHROME A* (*PHYA*), *GIGANTEA* (*GI*), *SHORT VEGETATIVE PERIOD* (*SVP*) and *AUXIN RESPONSE FACTOR* (*ARF*), were considered to be involved in the second floral initiation of the rebloomers.

**Conclusion:**

This research provides valuable information for the discovery of the reblooming-related genes. The insights into the molecular mechanisms of reblooming may accelerate the breeding of bearded iris and other perennials.

## Background

*Iris germanica* (bearded iris), characterized by the colorful beard on the three falls, is one of the most important perennials in the garden of spring, which can offer a full bloom from April to May [1, 2]. In the long breeding history, some reblooming bearded irises occurred, which could bloom for the second time in autumn of the same year [3–5]. The two flowering stages can lengthen the ornamental period by increasing the number rather than the length of florescence. Moreover, most rebloomers are blue and purple ones, which are rare in autumn-flowering plants. The more ornamental stages, the unique three falls and standards, the various flower colors and the ease of propagation, make the rebloomers increasingly popular in the market. However, the experience-oriented breeding of rebloomers is time-consuming and labor-wasting [1, 4]. Therefore, it is essential to explore the molecular mechanisms of reblooming bearded iris to reduce the breeding blindness and to create more reblooming cultivars.

The floral transition has been widely studied in the annual plant Arabidopsis, which is controlled by six main pathways [6, 7]. As for the reblooming perennials, temperature influenced the remontancy in the remontant octoploid strawberry (*Fragaria* × *ananassa*) [8], which could attribute to the temperature tolerance in the flower initiation of remontant strawberry [9]. In the study of remontant *Hydrangea macrophylla*, floral induction occurred under short-day (SD) and extended-day (ED) conditions but was more rapid under SD than ED [10]. Various studies have revealed the relationship between continuous flowering and the expression of *TERMINAL FLOWER1* (*TFL1*)*.* In *Fragaria vesca*, a 2-bp deletion in the coding region of the *TFL1* homologue introduced a frame shift and reversed the photoperiodic requirement for flowering, thus generating continuous flowering behavior [11, 12]. Similarly, the transcription of *TFL1* was blocked in continuous flowering roses due to an insertion of retrotransposon, and the absence of the floral repressor *TFL1* provoked the continuous blooming [11].

Meanwhile, the flowering pathways on reblooming bearded iris have been explored. For example, in a vernalization research about four reblooming bearded iris cultivars, only one cultivar, ‘Jennifer Rebecca’, was affected by vernalization and produced more inflorescences [13]. In another research, after the cold night treatment to the reblooming cultivar ‘Immortality’ and the spring-blooming cultivar ‘Arctic Fox’, only ‘Immortality’ developed flowers in anthesis. This research also hinted that the amount of cool night treatment may regulate floral initiation and development [14].

The previous researches mainly focused on the flowering pathways, which could unveil the reblooming mechanisms to some extent. However, from a more general perspective, reblooming may be due to the change of annual rhythms controlled by the circadian clock-related genes. It has been proved that circadian clock genes are involved in many biological processes, such as plant growth, development and physiology [15–19]. Flowering is an important output pathway of the circadian clock [20]. Some studies showed that the flower opening time was closely related to the daily oscillation of circadian rhythm genes [21–23]. Flower opening time reflects the daily rhythm of plants, and similarly, reblooming reflects the annual rhythm. Therefore, the change of annual circadian rhythm may engender the flowering out of season, for example, the reblooming in bearded iris, the remontant flowering in rose and the continuous flowering in strawberry. From this perspective, the analysis of circadian clock genes may give some clues to the understanding of reblooming.

In this research, we constructed a comprehensive transcriptome database of reblooming and once-blooming bearded iris, including six floral developmental stages. Our aim is to clarify the flowering genetic basis for these two types of bearded iris, thus providing valuable information regarding the molecular mechanisms of reblooming. The putative regulatory genes related to reblooming may be applied in the future molecular breeding of rebloomers, which could accelerate the breeding process. To the best of our knowledge, it is the first research revealing the molecular regulatory network of reblooming bearded iris.

## Results

### Construction of the sequencing hybrid population and phenotypical character analysis of hybrids

After the artificial emasculation and pollination of *I. germanica* ‘D’ and ‘H’, a total of 366 F_1_ seedlings were obtained.

In the F_1_ population, the flowering rate in spring was 65.23%, while the reblooming rate in autumn was 27.94%, suggesting that there existed once-bloomers and rebloomers in the same hybrid population (Additional file 1: Table S1). The identical parentage and different blooming characters made them the ideal plant materials to investigate the inheritance pattern of reblooming. Furthermore, the seed setting rate in autumn (37.50%) was larger than that of spring (30.00%), indicating a candidate approach to obtaining more hybrids in autumn.

The phenotypical performances of the F_1_ generation were different from those of their parents. In spring, the phenotypic values of LL (leaf length) and HF (height of individual flower) in F_1_ were larger than those of their parents, indicating that F_1_ had longer leaves and higher individual flowers (Table 1). However, the phenotypic value of NFS (number of flowers per scape) in spring was smaller than those of their parents, suggesting fewer flowers on an F_1_ stem. In the reblooming season (autumn), the phenotypic values of PH (plant height) and DF (diameter of flower) were smaller than those of their parents, the decrease of which may be owing to the comparatively cold climate in autumn. However, NLBS (number of leaves per blooming stem) of F_1_ in autumn was larger than those of parents, indicating that F_1_ need more nutrient accumulation to rebloom than their parents. Negative values of PV_s-a_ (difference between phenotypic value in spring and autumn) in NLBS and NFS revealed that F_1_ need more nutrient accumulation to rebloom in autumn than to bloom in spring. As a result, F_1_ could generate more flowers on a stem in autumn than in spring.

**Table 1:** The phenotypic values in spring and autumn for the ten phenotypic traits of *Iris germanica* ‘D’ × *I. germanica* ‘H’

Characters	PV_D_	PV_H_	PV_s_	PV_a_	PV_s-a_
PH / cm	63.56 ± 7.32ab	70.80 ± 5.29a	58.34 ± 16.22ab	52.82 ± 11.07b	5.52
LL / cm	40.68 ± 2.72b	47.80 ± 2.17ab	49.81 ± 10.01a	40.06 ± 6.49b	9.75
NLBS	2.34 ± 0.61b	2.68 ± 0.46b	2.08 ± 0.28b	5.21 ± 1.10a	−3.13
NLNS	5.24 ± 0.83a	5.78 ± 0.86a	4.72 ± 1.67a	4.64 ± 0.91a	0.08
NS	3.56 ± 0.61a	3.12 ± 0.52a	4.15 ± 1.70a	3.04 ± 0.64a	1.11
NFS	6.20 ± 0.84a	4.25 ± 1.09bc	3.63 ± 1.24c	5.46 ± 1.86ab	−1.83
HF / cm	9.80 ± 0.95b	11.20 ± 0.58ab	12.02 ± 1.49a	9.81 ± 1.69b	2.21
DF / cm	14.66 ± 0.76ab	15.40 ± 0.89a	14.65 ± 1.58ab	13.28 ± 1.69b	1.37
LF / cm	8.70 ± 0.46a	8.52 ± 0.35a	8.90 ± 0.87a	8.74 ± 0.90a	0.16
WF / cm	7.02 ± 0.97b	7.84 ± 0.21a	6.86 ± 0.76b	6.76 ± 0.81b	0.10

*PH* plant height, *LL* leaf length, *NLBS* number of leaves per blooming stem, *NLNS* number of leaves per non-blooming stem, *NS* number of stems, *NFS* number of flowers per scape, *HF* height of the individual flower, *DF* diameter of flower, *LF* length of fall, *WF* width of fall, *PV*_*D*_ phenotypic value of *I. germanica* ‘D’, *PV*_*H*_ phenotypic value of *I. germanica* ‘H’, *PV*_*s*_ phenotypic value of spring, *PV*_*a*_ phenotypic value of autumn, *PV*_*s-a*_ difference between phenotypic value in spring and autumn

### RNA extraction, RNA-seq and assembly

Ten libraries of total RNA were used for high throughput sequencing (Table 2, Additional file 2: Fig. S1). After trimming the raw data, a total of 428,442,355 clean reads from RB (rebloomers) transcriptome and 307,674,756 clean reads from OB (once-bloomers) transcriptome were obtained (Table 3). The Q30 percentages for RB and OB transcriptome were 89.00 and 90.36%, respectively. In addition, the GC contents were 50.49 and 50.47% for RB and OB transcriptome, respectively, indicating the high quality of the sequencing data.

**Table 2:** The sampled stages and sampled blooming types of transcriptome sequencing

Stages	The floral initiation stage of spring flowering (T1)	The stage after entering dormancy (T2)	The stage after dormancy release (T3)	The bud stage of spring flowering (T4)	The floral initiation stage of autumn flowering (T5)	The bud stage of autumn flowering (T6)
Rebloomers (RB)	RB-T1	RB-T2	RB-T3	RB-T4	RB-T5	RB-T6
Once-bloomers (OB)	OB-T1	OB-T2	OB-T3	OB-T4		

Note: The samples of T1, T2, T3 and T5 were composed of apical meristem and leaves. The samples of T4 and T6 were composed of flower buds and leaves

**Table 3:** Summary of Illumina transcriptome sequencing

	RB Transcriptome	OB Transcriptome	Total Transcriptome
Number of raw reads	445,275,859	317,078,510	762,354,369
Number of clean reads	428,442,355	307,674,756	736,117,111
Total nt of raw reads (G)	133,582,757,700	95,123,553,000	228,706,310,700
Total nt of clean reads (G)	128,076,667,900	91,976,233,960	220,052,901,900
Number of bases	128,076,667,942	91,976,233,956	220,052,901,898
GC percentage (%)	50.49	50.47	50.48
Q30 percentage (%)	89.00	90.36	89.55

*RB* rebloomers, *OB* once-bloomers

After the assembly of the clean reads, a total of 100,391 unigenes were obtained, and the mean length was 785 bp (Additional file 1: Table S2). The N50 of the assembled unigenes was 1368 bp. For transcripts, a total of 203,398 transcripts with the mean length being 985 bp were obtained, the N50 of which was 1533 bp. The percentage of unigenes with sequence size between 200 bp and 300 bp was the largest, narrowly followed by those between 300 bp and 400 bp (Additional file 2: Fig. S2). The above-mentioned data showed the high quality of the assembled unigenes.

### Functional annotation of unigenes

A total of 49,824 unigenes were annotated against the five public databases, including NCBI Non-redundant protein sequences (Nr), Gene Ontology (GO), Clusters of Orthologous Groups (COG), Kyoto Encyclopedia of Genes and Genomes (KEGG) and Swiss-Prot. The numbers of annotated unigenes in Nr, GO, COG, KEGG and Swiss-prot were 46,783, 29,310, 14,063, 19,071 and 30,697, respectively (Additional file 1: Table S3).

**Fig. 1:**
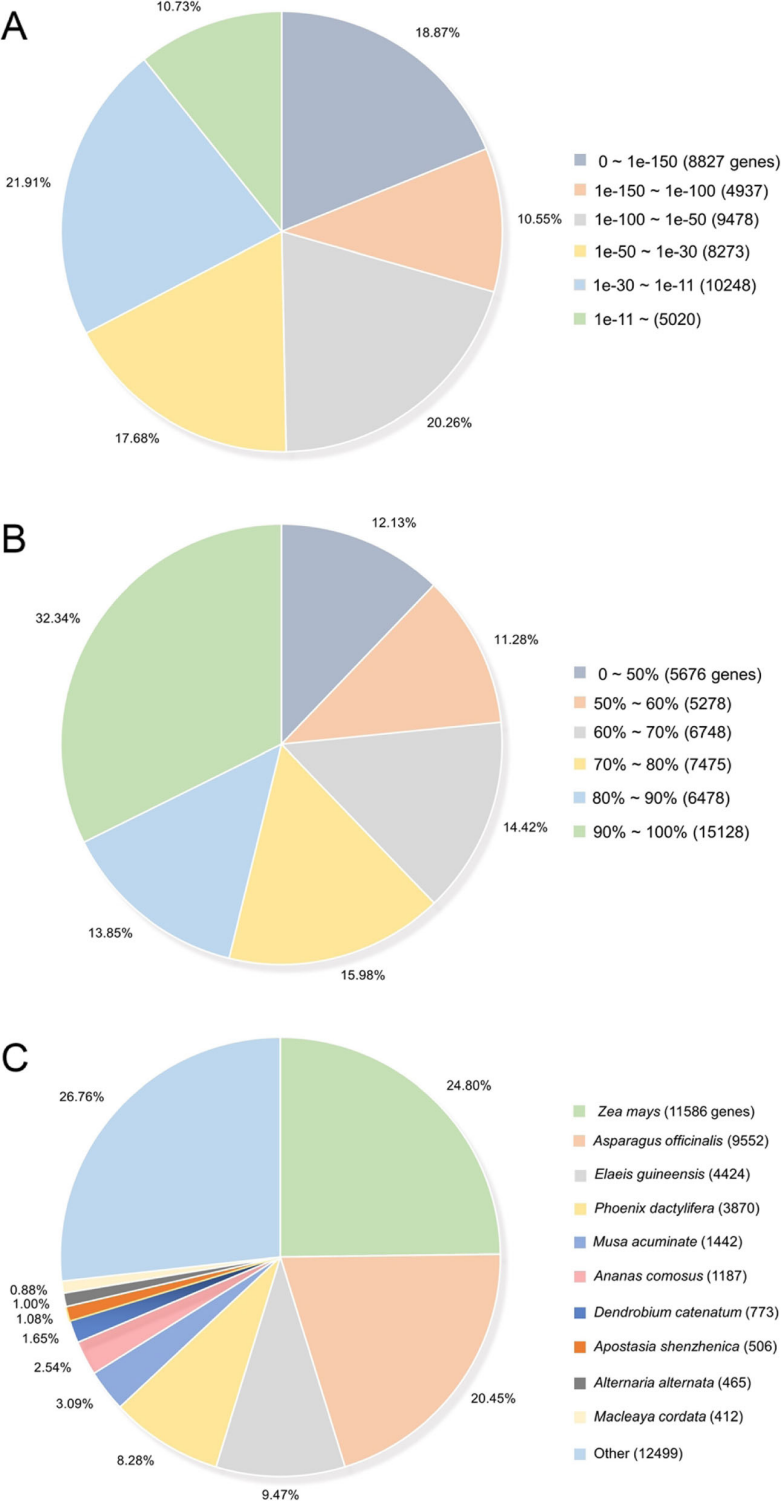
Characteristics of the iris unigenes against the NCBI non-redundant protein sequences (Nr) database. (A) E-value distribution of BLAST hits for each unigene with an E-value cutoff of 1.0E-11. (B) Similarity distribution of the top BLAST hits for each unigene. (C) Species distribution of the top BLAST hits

According to the E-value distribution of BLAST hitting against the Nr database, 49.68% of the matched sequences showed high homologies with the E-value < 1.0E-50, while 50.32% of them had E-value > 1.0E-50 (Fig. 1A). The similarity distribution of the top BLAST hits showed that 46.19% of the matched sequences were in similarity larger than 80% (Fig. 1B). The top 3 species sharing homologies with *I. germanica* were *Zea mays* (24.80%), *Asperagus officinalis* (20.45%) and *Elaeis guineensis* (9.47%) (Fig. 1C). All these three species are monocots, the same as *I. germanica*, suggesting that the assembly and annotations of the iris RB and OB transcriptome are correct and reliable.

**Fig. 2:**
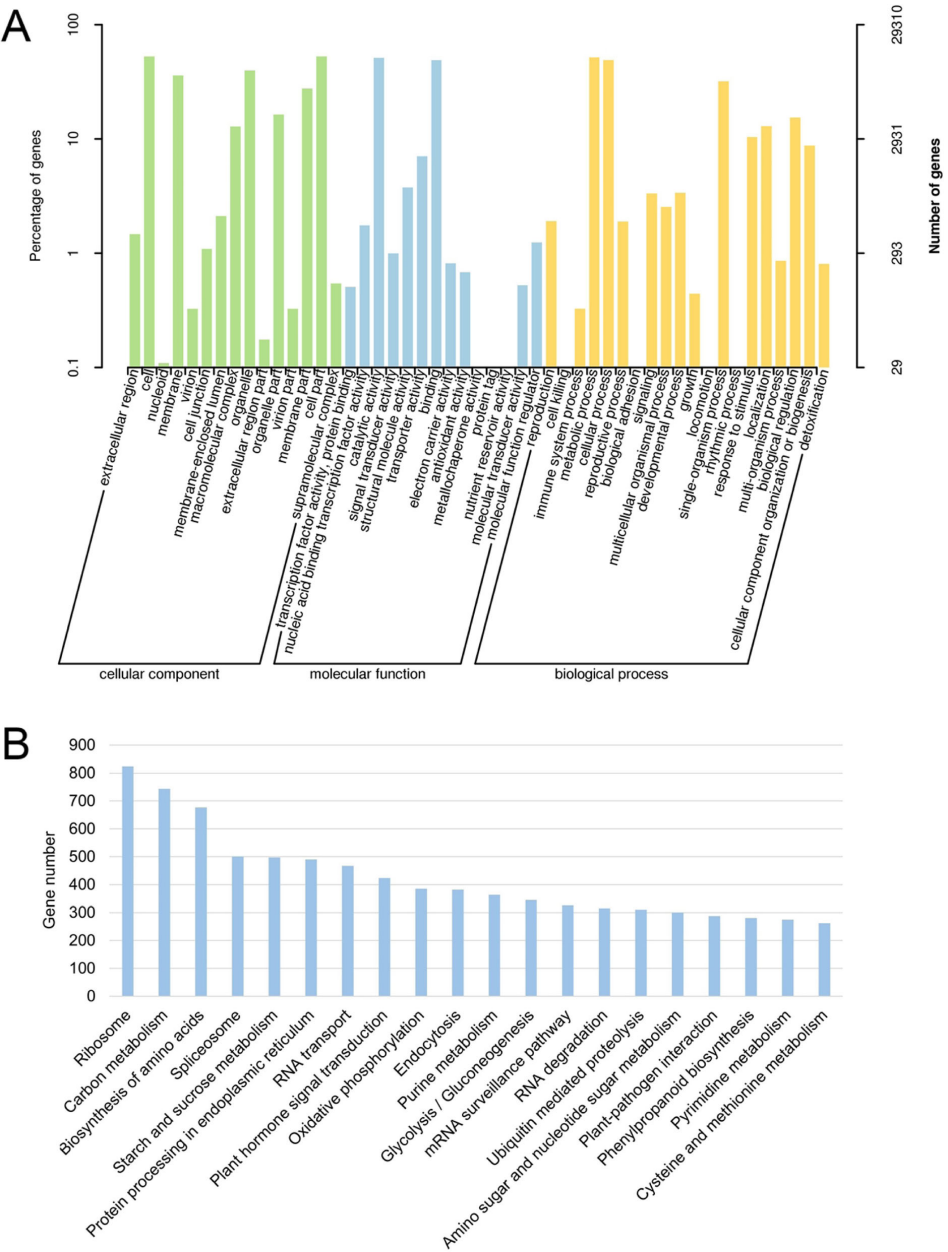
GO and KEGG functional analysis of all unigenes. (A) GO functional classification of all the unigenes. The left-hand y-axis indicates the percentage of the annotated unigenes. The right-hand y-axis indicates the number of the annotated unigenes. (B) The assignment of *I. germanica* unigenes to the top 20 KEGG biological pathways

In the GO analysis, 29,310 unigenes were assigned to 42 GO terms under three categories (Fig. 2A, Additional file 1: Table S3). High percentage of unigenes were assigned into “cell”, “cell part”, “organelle” and “membrane” under the “cellular component” category. The proportions of “catalytic activity” and “binding” were the largest in the “molecular function” category. “Metabolic process” and “cellular process” occupied the most under the “biological process” category (Fig. 2A, Additional file 1: Table S4).

A total of 19,071 unigenes were annotated against the KEGG database and were assigned to 129 KEGG pathways (Additional file 1: Table S3 and Table S5). It is worth mentioning that “plant hormone signal transduction” was among the top 20 KEGG pathways, which was closely related to flowering (Fig. 2B). These top 20 pathways provide a valuable reference for investigating the specific processes, functions and pathways during *I. germanica* flower development.

### Functional annotation of differentially expressed genes (DEGs)

**Fig. 3:**
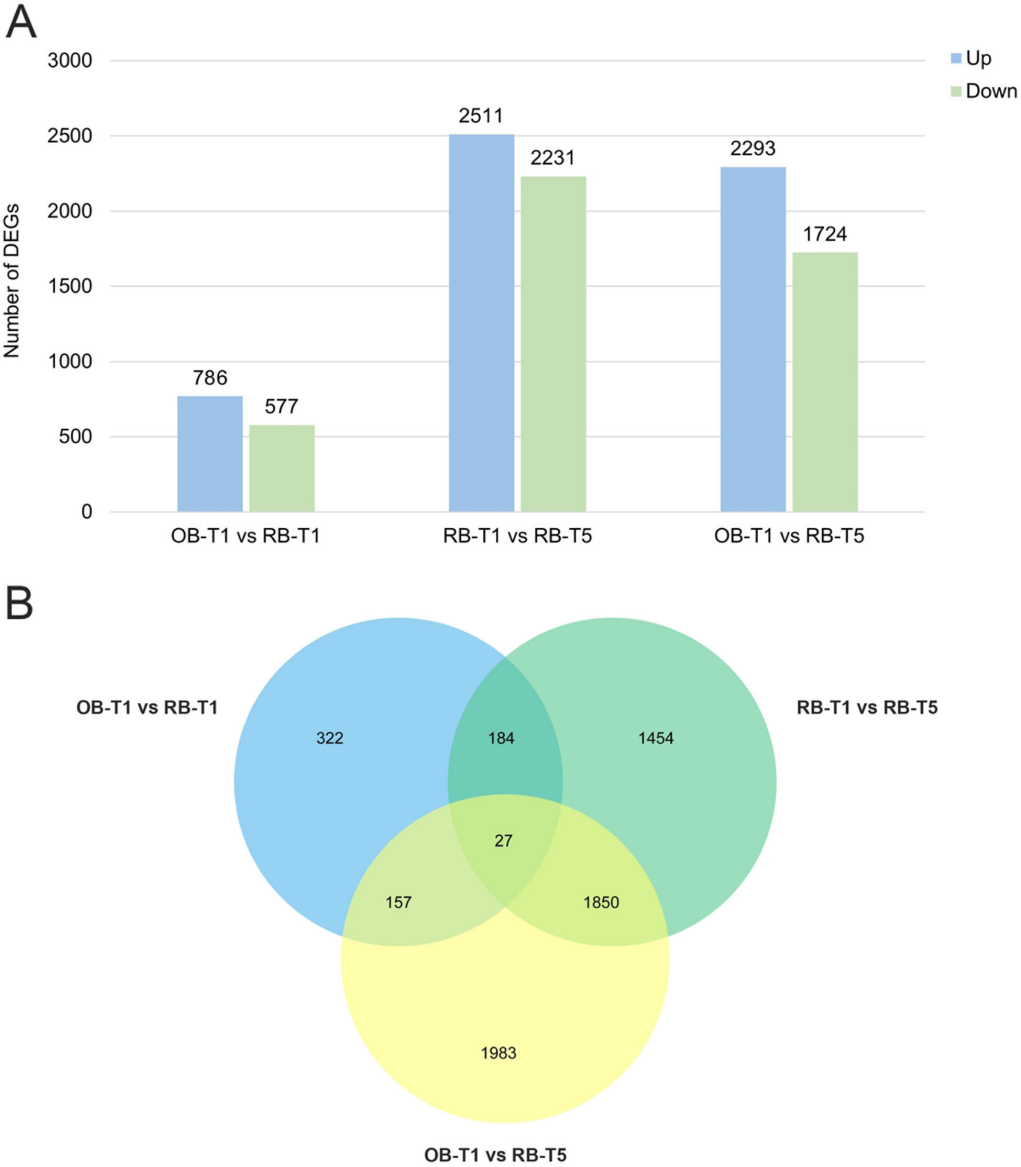
DEGs among the different developmental stages. (A) The numbers of DEGs in different comparisons, including OB-T1 vs RB-T1, RB-T1 vs RB-T5 and OB-T1 vs RB-T5. (B) Venn diagram of the numbers of unique and common DEGs among the comparisons between OB-T1 vs RB-T1, RB-T1 vs RB-T5 and OB-T1 vs RB-T5

DEGs were filtered from the comparison of three stages, including the floral initiation stage of spring flowering in once-bloomers (OB-T1), the floral initiation stage of spring flowering (RB-T1) and autumn flowering (RB-T5) in rebloomers. A total of 690, 3515 and 2941 DEGs were annotated against the five public databases (Nr, GO, COG, KEGG and Swiss-prot), from the comparison of OB-T1 vs RB-T1, RB-T1 vs RB-T5 and OB-T1 vs RB-T5, respectively (Table 4). In the comparison of OB-T1 vs RB-T1, a total of 1363 genes were differentially expressed (786 up-regulated and 577 down-regulated). The numbers of DEGs in RB-T1 vs RB-T5 and OB-T1 vs RB-T5 were 4742 (2511 up-regulated and 2231 down-regulated) and 4017 (2293 up-regulated and 1724 down-regulated), respectively (Fig. 3a, Additional file 1: Table S6, Additional file 2: Fig. S3). Among these DEGs, some genes may regulate the spring blooming and autumn reblooming. Furthermore, in the Venn diagram, a total of 27 DEGs existed in all the three comparisons (Fig. 3b).

**Table 4:** Statistical analysis of DEG annotation in different comparisons

Type	OB-T1 vs RB-T1	RB-T1 vs RB-T5	OB-T1 vs RB-T5
COG	196	1079	1074
GO	331	1958	1895
KEGG	210	1636	2175
Swiss-prot	491	2594	2530
Nr	682	3442	2859
All annotated	690	3515	2941

### KEGG pathway and GO enrichment analysis of DEGs

The DEGs were annotated against the KEGG and GO databases, in order to further identify their biological functions. The assigned functions of DEGs covered a wide range of KEGG and GO terms, indicating that the transcriptome data represented a broad variety of transcripts in *I. germanica*.

**Fig. 4:**
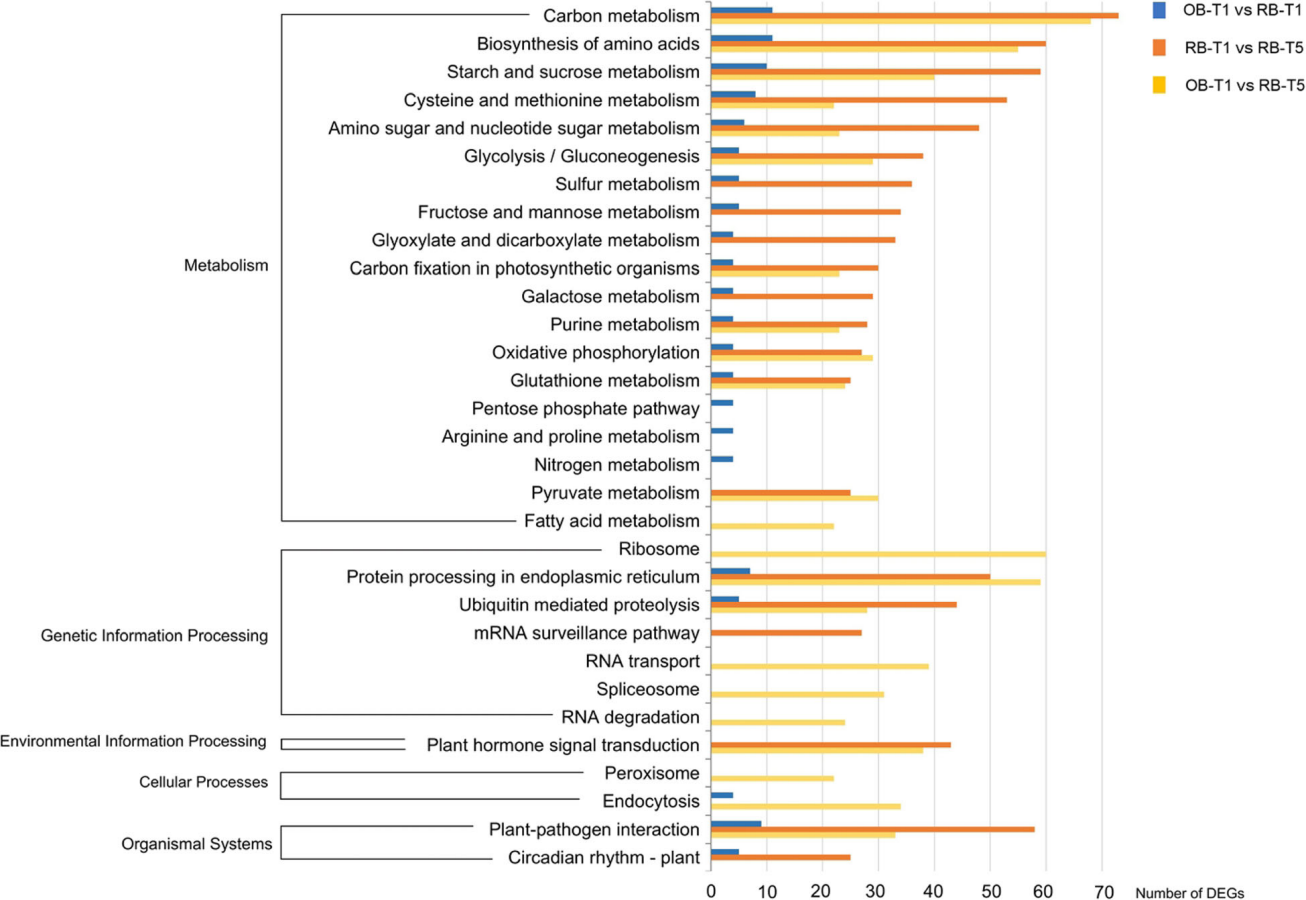
The top 20 enriched KEGG pathways of the DEGs after the comparisons of OB-T1 vs RB-T1, RB-T1 vs RB-T5 and OB-T1 vs RB-T5

In the KEGG pathway analysis, we mainly focused on the “plant hormone signal transduction” under the “environmental information processing” category and the “circadian rhythm-plant” under the “organismal systems” category, which were closely related to the flowering of plants. In the comparison of OB-T1 vs RB-T1, two DEGs were annotated into the “plant hormone signal transduction” pathway, and five DEGs were annotated into the “circadian rhythm-plant” pathway (Additional file 1: Table S7, Additional file 2: Fig. S4). In the comparison of RB-T1 vs RB-T5, the numbers of DEGs annotated into “plant hormone signal transduction” pathway and “circadian rhythm-plant” pathway were 43 and 25, respectively (Additional file 1: Table S8, Additional file 2: Fig. S4). Similarly, in the comparison of OB-T1 vs RB-T5, the numbers of DEGs annotated into “plant hormone signal transduction” pathway and “circadian rhythm-plant” pathway were 38 and 20, respectively (Additional file 1: Table S9, Additional file 2: Fig. S4). In addition, these two pathways were listed in the top 20 enriched KEGG pathways (Fig. 4), which supported that the DEGs in these two pathways were likely to play essential roles in reblooming.

**Fig. 5:**
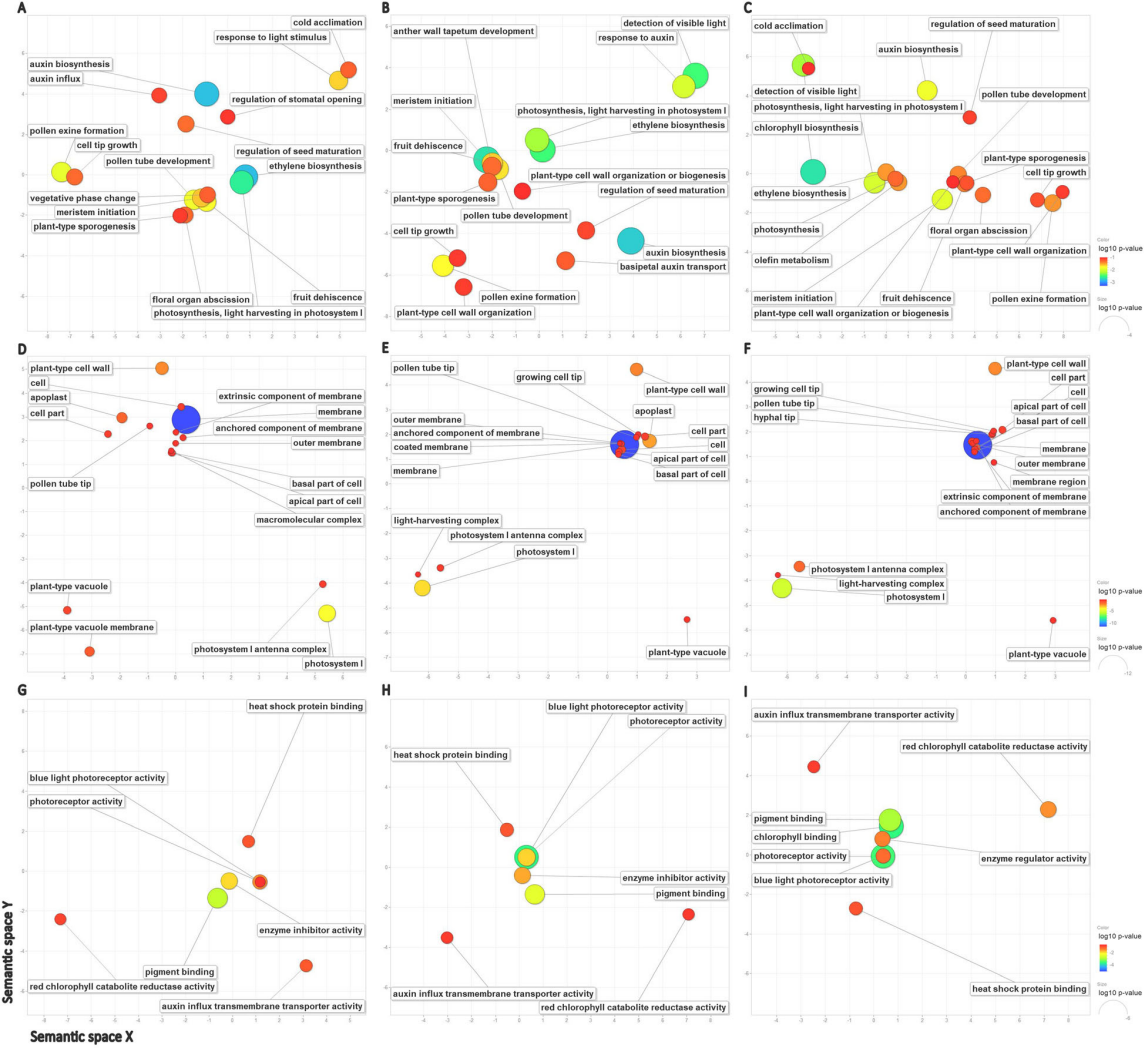
GO pathway analysis for DEGs in the three comparisons (OB-T1 vs RB-T1, RB-T1 vs RB-T5, OB-T1 vs RB-T5) by using REVIGO. (A, B & C) represent Biological Process (BP) in OB-T1 vs RB-T1, RB-T1 vs RB-T5 and OB-T1 vs RB-T5, respectively; (D, E & F) represent Cellular Components (CC) in OB-T1 vs RB-T1, RB-T1 vs RB-T5 and OB-T1 vs RB-T5, respectively; (G, H & I) represent Molecular Function (MF) in OB-T1 vs RB-T1, RB-T1 vs RB-T5 and OB-T1 vs RB-T5, respectively. Bubble color represents the log10 (*P*-value) for false discovery rates, as shown in the scale on the right. The larger bubble size indicates the enriched GO terms

In the GO analysis of DEGs from the three comparisons (OB-T1 vs RB-T1, RB-T1 vs RB-T5, OB-T1 vs RB-T5), “metabolic process” was the most enriched GO terms in the category of “biological process”; “cell” was the most annotated in the “cellular component” category; “catalytic activity” was most abundant in the “molecular function” category (Additional file 1: Table S10, Additional file 2: Fig. S5). The redundant GO terms were removed and the GO differences between the three comparisons were visualized in the Reduce and Visualize GO (REVIGO) analysis. The GO terms in the “biological process” part, such as photosynthesis, light harvesting in photosystem I (GO:0009768), auxin biosynthesis (GO:0009851), and meristem initiation (GO:0010014), were highly enriched in all the three comparisons (Fig. 5A-C). Cold acclimation (GO:0009631) and detection of visible light (GO:0009584) were highly enriched in two of the comparisons. Similarly, photosystem I antenna complex (GO:0009782), photosystem I (GO:0009522) and light-harvesting complex (GO:0030076) in the “cellular component” part (Fig. 5D-F), and blue light photoreceptor activity (GO:0009882) and photoreceptor activity (GO:0009881) in the “molecular function” part (Fig. 5G-I), were enriched in all the three comparisons. It is reasonable to conclude that the formation of reblooming may be related to light sensing, cold acclimation and growth speed.

### The circadian rhythm related genes and photoperiod pathway genes may regulate reblooming

The circadian clock is the mastermind of the plant life, since it has been proved to be involved in plant growth, development and physiology. Circadian clock provides not only time prediction features, but also adaptability to the changing environmental conditions [16–19]. Reblooming, as an important developmental event, may be influenced by the expression profile of the circadian rhythm related genes. Therefore, the photoperiod related genes, as one of the output pathways of the circadian clock, may participate in the formation of reblooming.

**Fig. 6:**
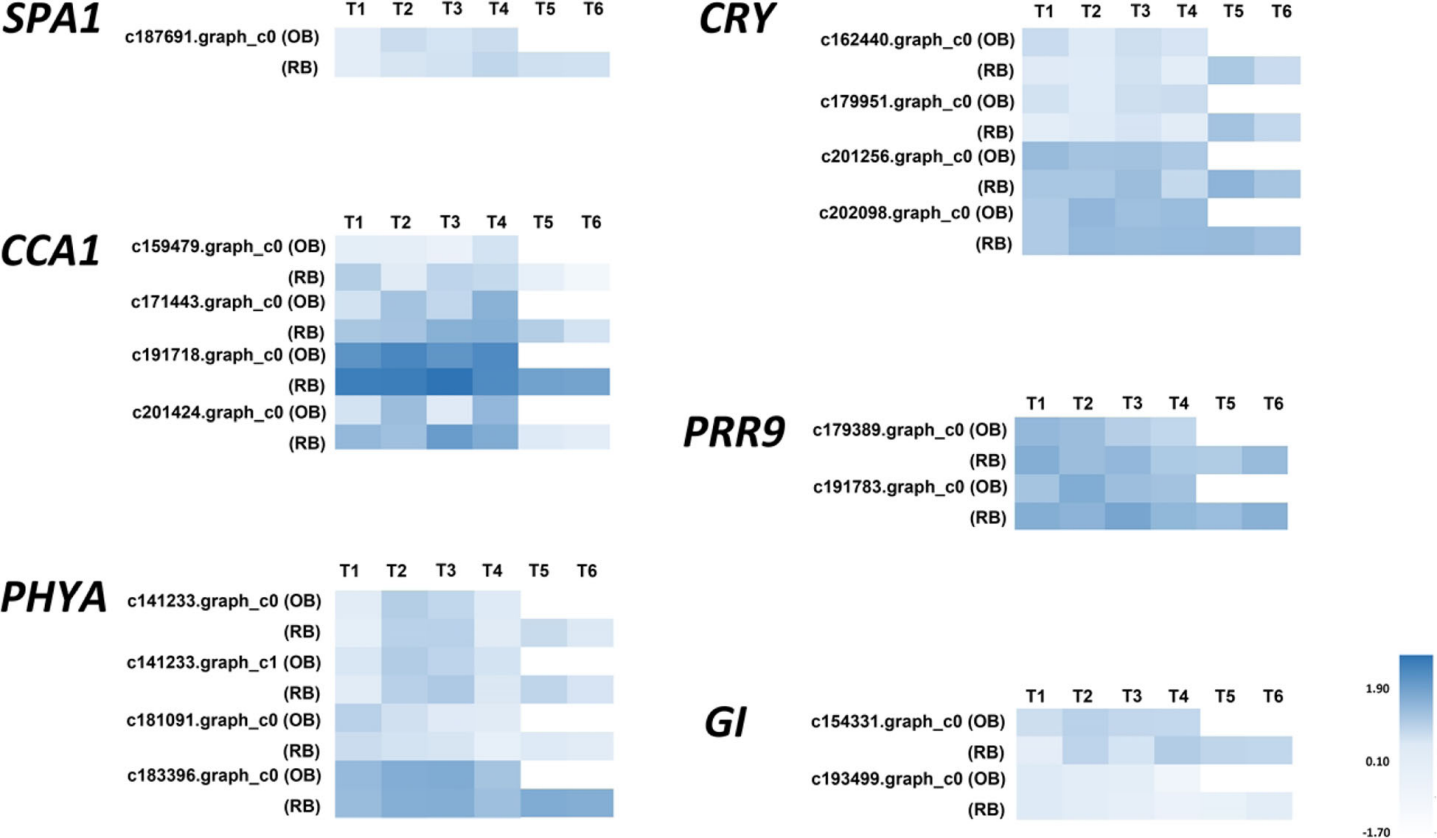
The heat map of DEGs in photoperiod pathway. Dark-blue indicates a relative increase of expression, and light-blue represents a relative decrease of expression. *SPA1*: *SUPPRESSOR OF PHYTOCHROME A1*; *CCA1*: *CIRCADIAN CLOCK ASSOCIATED 1*; *PHYA*: *PHYTOCHROME A*; *CRY*: *CRYPTOCHROME*; *PRR9*: *PSEUDO-RESPONSE REGULATOR 9*; *GI*: *GIGANTEA*; OB: once-bloomers; RB: rebloomers. T1-T6 represents the six developmental stages in Table 2

In the comparison of the two floral initiation stages in rebloomers (RB-T1 vs RB-T5), *SUPPRESSOR OF PHYTOCHROME A1* (*SPA1*)*, PHYTOCHROME A* (*PHYA*), *CRYPTOCHROME* (*CRY*) and *GIGANTEA* (*GI*) were significantly up-regulated in RB-T5 compared to RB-T1 (Fig. 6). In contrast, *PSEUDO-RESPONSE REGULATOR 9* (*PRR9*) and *CIRCADIAN CLOCK ASSOCIATED 1* (*CCA1*) were significantly down-regulated in RB-T5 compared to RB-T1. Moreover, in the comparison of the two floral initiation stages in once-bloomers and rebloomers (OB-T1 vs RB-T5), *CCA1*, *GI*, *SPA1*, *PHYA* and *CRY* were significantly up-regulated. However, no gene was significantly down-regulated in OB-T1 vs RB-T5.

### The temperature insensitivity engenders more than one floral initiations

Both once-bloomers and rebloomers could initiate the first floral transition in late October every year. For once-bloomers, only after the vernalization in winter could the floral buds complete the following development and bloom in May. However, the rebloomers could initiate a second floral transition in early June and bloom in October, without vernalization. Therefore, the temperature sensitivity may be different in rebloomers and once-bloomers, and the vernalization related genes may have some influence on the formation of reblooming.

The vernalization pathway genes, *PHOTOPERIOD-INDEPENDENT EARLY FLOWERING 1* (*PIE1*), *FRIGIDA* (*FRI*) and *VERNALIZATION INSENSITIVE 3* (*VIN3*) were considered as DEGs (Additional file 2: Fig. S6). The expression of the floral repressor *FLOWERING LOCUS C* (*FLC*) was regulated by *FRI*, *VIN3* and *PIE1*. However, the absence of *FLC* in DEGs indicated that its expression was relatively stable during the floral development, probably due to the interactions of its upstream genes canceling each other out. As a result, the temperature fluctuation had little effect on the floral initiation and development, which facilitated the second floral initiation and floral bud development in summer.

### The fast growth speed facilitates the twice blooming in a year

Unlike the once-bloomers, rebloomers could complete the cycles from vegetative to reproductive growth twice a year. The rebloomers initiated their second floral transition in early June and could bloom in October. The shortened vegetative period indicates the faster growth speed. Therefore, some DEGs relating to vegetative growth rate may regulate the formation of reblooming.

**Fig. 7:**
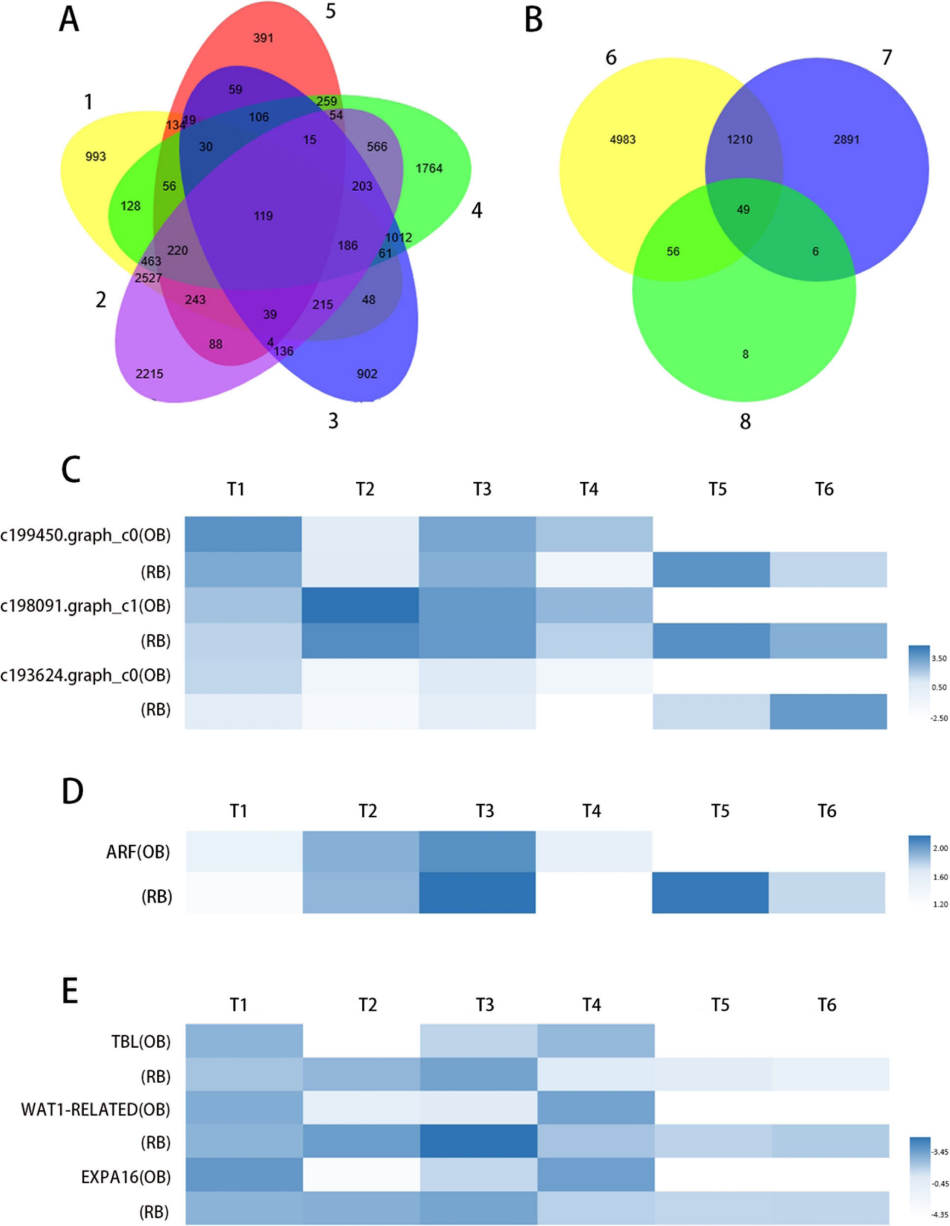
The analysis of growth related genes. (A) The Venn diagram of two adjacent stages of rebloomers. “1” represents the DEGs in RB-T4 vs RB-T5; “2” represents the DEGs in RB-T3 vs RB-T4; “3” represents the DEGs in RB-T1 vs RB-T2; “4” represents the DEGs in RB-T6 vs RB-T1; “5” represents the DEGs in RB-T5 vs RB-T6. (B) The Venn diagram of two adjacent stages of rebloomers and once-bloomers. “6” represents the DEGs in OB-T1 vs OB-T2; “7” represents the DEGs in OB-T3 vs OB-T4; “8” represents the 119 DEGs in “1”-“5” of Fig. 7A. (C) The heat map of DEGs annotated into the “starch and sucrose metabolism” pathway in KEGG database. (D) The heat map of *AUXIN RESPONSE FACTOR* (*ARF*). (E) The heat map of DEGs related to cell wall function. Dark-blue indicates a relative increase of expression, and light-blue represents a relative decrease of expression. T1-T6 represents the six developmental stages in Table 2. OB and RB represent once-bloomers and rebloomers, respectively

Among all the 70 putative DEGs from the comparison between two adjacent sampling periods (Fig. 7a-b), three DEGs were categorized into the “starch and sucrose metabolism” pathway in the KEGG database (Fig. 7c). Genes in this pathway regulated the metabolism of starch and sucrose, which were the fundamental materials for vegetative growth. Besides, one *AUXIN RESPONSE FACTOR* (*ARF*) was detected to differentially express in all the comparisons of two adjacent periods in rebloomers, but was absent in the counterparts of once-bloomers (Fig. 7d). The vegetative growth between the bud stage of spring flowering (T4) and the floral initiation stage of autumn flowering (T5) was important for rebloomers. In rebloomers, the expression level of *ARF* in T5 was much higher than T4, making it a candidate key gene to accelerate the vegetative growth. Besides, three DEGs related to cell wall function were filtered out (Fig. 7e), including *TRICHOME BRIRFRINGENCE-LIKE (TBL), WAT1-RELATED* and *EXPANSIN-A16-LIKE (EXPA16)*. The expression level of these three genes in RB-T2 (the stage after entering dormancy in rebloomers) was higher than RB-T1, while OB-T2 (the stage after entering dormancy in once-bloomers) was lower than OB-T1. This indicated that the cell wall function was still active for rebloomers before entering dormancy, but weak in once-bloomers. The active cell wall function guaranteed the rapid growth rate of rebloomers, making it possible to complete two cycles of flowering in a single year.

### The putative regulatory network of reblooming

**Fig. 8:**
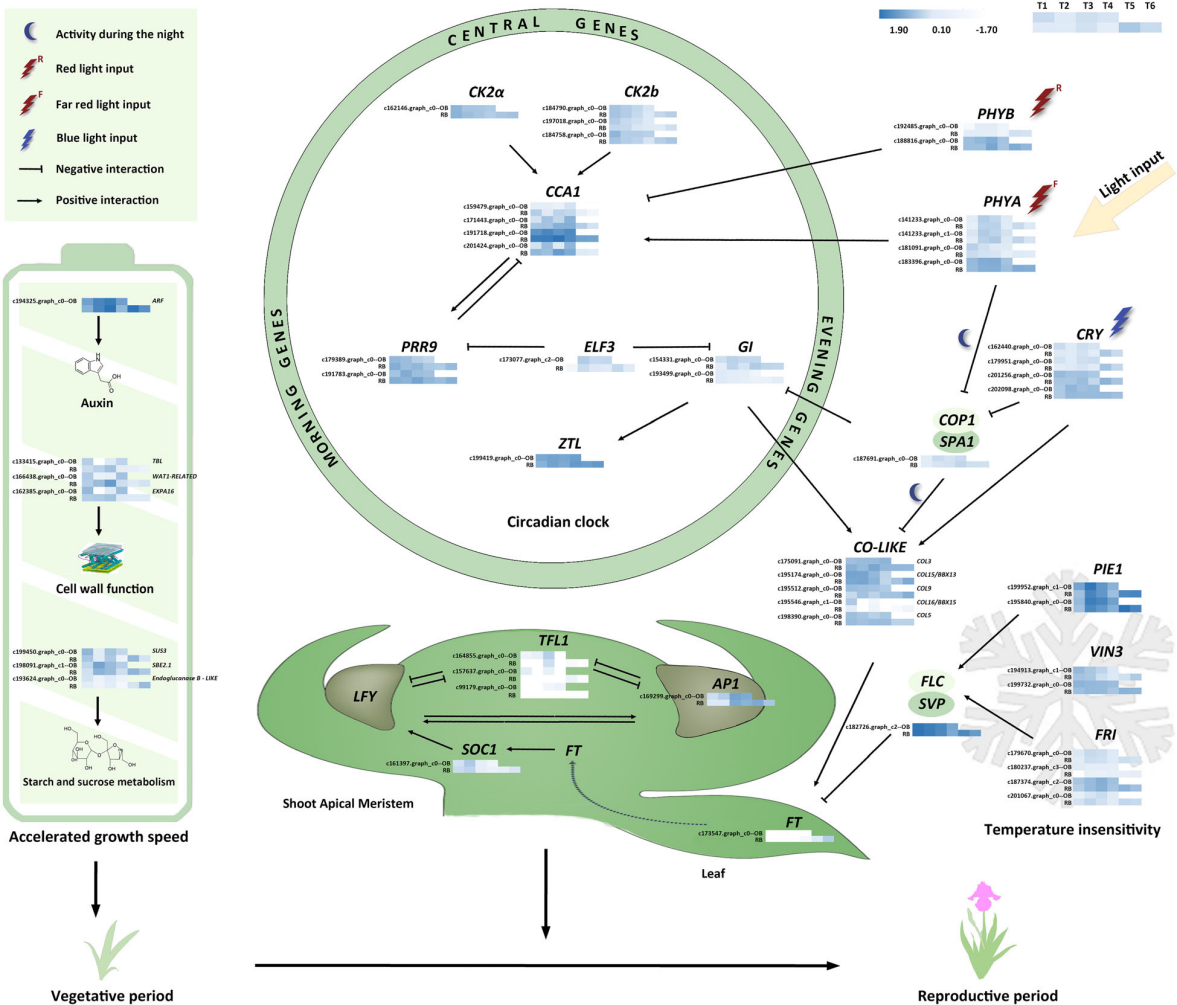
The putative floral development regulatory network of reblooming bearded iris. This regulatory network was modified and integrated from the transcriptome sequencing results of the circadian rhythm related genes, the flowering pathway (including vernalization and photoperiod pathways) genes and the growth related pathway genes. Dark-blue indicates a relative increase of expression, and light-blue represents a relative decrease of expression. T1-T6 represents the six developmental stages in Table 2. OB and RB represent once-bloomers and rebloomers, respectively

Receiving the signals from the upstream genes of the circadian rhythm regulatory network, photoperiod pathway, vernalization pathway and the growth related pathway, the flowering regulators would integrate these signals and exert some influence on the flowering. *SHORT VEGETATIVE PERIOD* (*SVP*), an important flowering suppressor, was identified as a DEG in the comparison of RB-T1 vs RB-T5 (Fig. 8). In rebloomers, the expression level of *SVP* in the second floral initiation period (RB-T5) was much lower than that of the first one (RB-T1), thus fascinating the second floral initiation. However, *TFL1*, another flowering repressor controlling the remontant flowering in rose and strawberry [11, 12, 24], showed a higher expression level in the second floral initiation period, the reason of which need further investigation. In spite of this, the expression of two flowering promoters *APETALA 1* (*AP1*) and *FLOWERING LOCUS T* (*FT*) in RB-T5 were enhanced compared to T1, which was the direct reason for reblooming.

The protein-protein interactions among the putative regulatory genes in Fig. 8 were analyzed in the STRING database (Additional file 2: Fig. S7A). In the hub gene analysis by Cytoscape, *GI* was identified as a hub gene that interacted with all the top 10 interaction nodes (Additional file 2: Fig. S7B). Besides, *PHYA* and *FT* were among the top 10 interaction nodes. Therefore, *GI*, *PHYA* and *FT* may be key regulators in the development of flowers and played an essential role in the formation of rebloomers.

### **Verification of RNA-seq results by** qRT-PCR

**Fig. 9:**
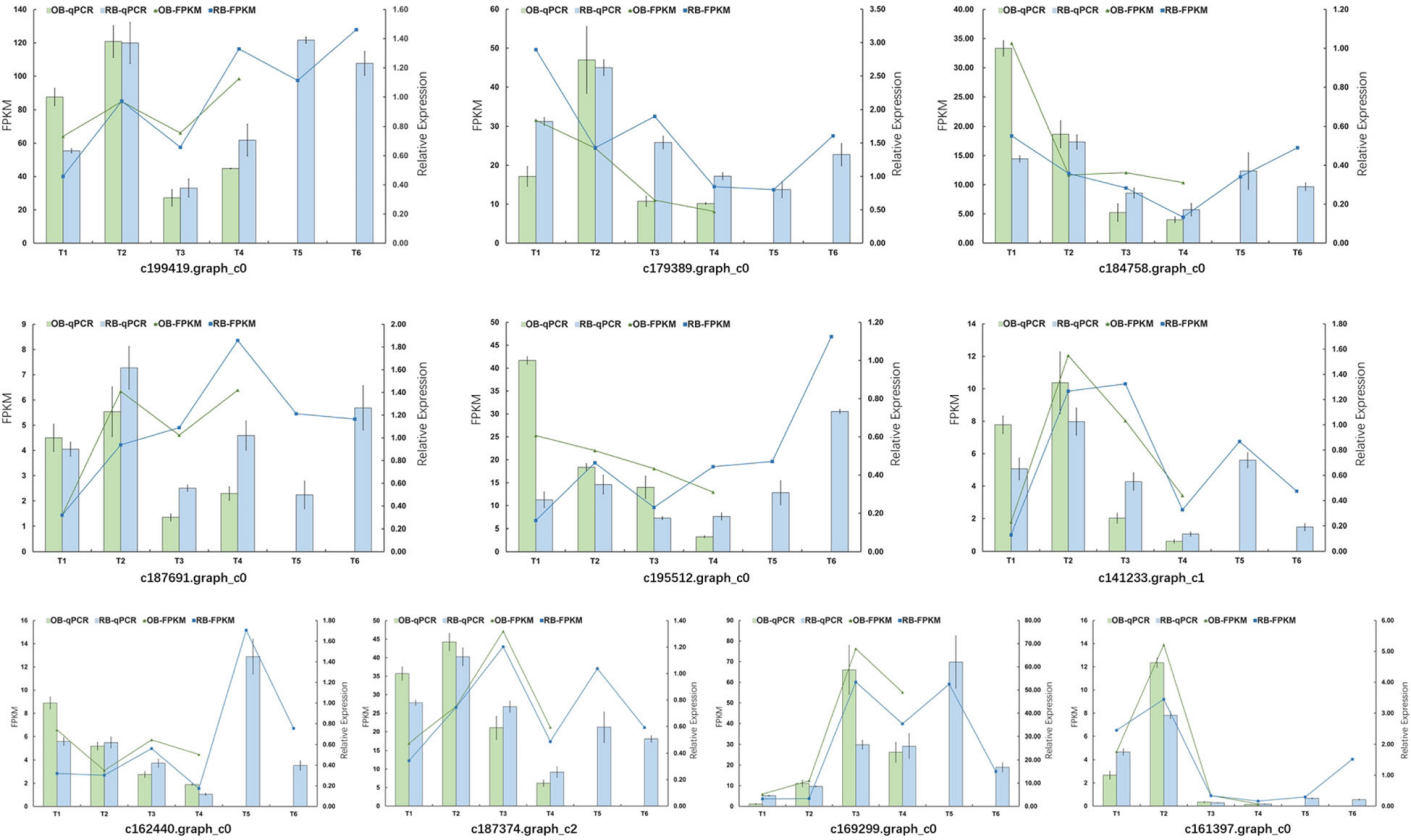
The expression profiles of the ten DEGs. The expression patterns of *ZEITLUPE* (*ZTL*: c199419.graph_c0), *PSEUDO-RESPONSE REGULATOR 9* (*PRR9*: c179389.graph_c0), *CASEIN KINASE 2α* (*CK2α*: c184758.graph_c0), *SUPPRESSOR OF PHYTOCHROME A1* (*SPA1*: c187691.graph_c0), *CONSTANS* (*CO*: c195512.graph_c0), *PHYTOCHROME A* (*PHYA*: c141233.graph_c1), *CRYPTOCHROME* (*CRY*: c162440.graph_c0), *FRIGIDA* (*FRI*: c187374.graph_c2), *APETALA 1* (*AP1*: c169299.graph_c0) and *SUPPRESSOR OF OVEREXPRESSION OF CONSTANS 1* (*SOC1*: c161397.graph_c0) were determined by qRT-PCR (relative expression compared to stage T1) and RNA-seq analysis (FPKM values). Error bars represent the standard deviation of the means. T1-T6 represents the six developmental stages in Table 2. OB and RB represent once-bloomers and rebloomers, respectively

To verify the reliability of the transcriptome sequencing data, we selected ten flowering-related DEGs randomly and performed qRT-PCR using the same samples as those in transcriptome sequencing. The iris *ACTIN* gene was used as the endogenous reference gene. The results showed that the expression levels of the selected genes were basically consistent with the RNA-seq results (Fig. 9), which confirmed the validity of the de novo assembled transcriptome data and the following analysis.

## Discussion

### The construction of a hybrid population is an essential step for effectively detecting key genes of reblooming

Transcriptome profiling is an effective way to detect DEGs from various sequencing groups, thereby providing clues to the detection of key genes involved in the target characters. For example, the key genes regulating the reblooming characteristics could be detected by sequencing the reblooming and once-blooming plants, respectively. However, due to the different genetic backgrounds, a large number of DEGs related to other characteristics could be detected, such as flower color, drought tolerance, etc. Consequently, it would lead to the complexity of key gene screening. In order to handle this problem, the two sequencing groups should have an extremely similar genetic background, with only different blooming characteristics.

Hybridization could shed much light on this problem. We used two cultivars (*I. germanica* ‘D’ and *I. germanica* ‘H’) as parents to generate the F_1_ generation including reblooming individuals and once-blooming ones. After that, comparative transcriptome analysis was carried out on the reblooming and once-blooming offspring, respectively. Due to their identical parentage, the detected DEGs were much fewer and were more likely to be associated with reblooming. The innovative sequencing of F_1_ individuals could reduce the number of DEGs, thus providing convenience for the subsequent key genes screening.

Besides, F_1_ had longer leaves, higher individual flowers, but fewer flowers than their parents (Table 1), which provided more diversity of the phenotypic variations. The larger seed setting rate in autumn indicated a candidate way to obtain more hybrids than that in spring (Additional file 1: Table S1).

### Illumina sequencing and unigene annotation provide comprehensive transcriptome information of *I. germanica*

*I. germanica* is an essential ornamental perennial in the spring garden, and the reblooming ones can provide diversity to the autumn landscape. Even though the continuous flowering is well investigated in rose [11, 25, 26], woodland strawberry [27, 28] and longan [29], little is known about the mechanisms of the reblooming bearded iris. In this study, a total of 100,391 unigenes were obtained by Illumina sequencing of flower buds, apical meristem and leaves from six floral development stages, among which 49,824 unigenes (49.63% of 100,391) could be annotated against the public databases (Additional file 1: Table S2 and Table S3). Our annotation percentage of the assembled unigenes was similar to the other transcriptome profiling of *I. halophile* (47.57%) [30] and *I. lactea* var. *chinensis* (46.57%) [31]. The annotation rate could be improved if the genomic information is available in the future.

### DEG analysis may provide some references for key gene screening

We removed the redundant GO terms and visualized the GO differences among different groups by the REVIGO online tool. The analysis of the most enriched GO terms in the three categories showed that most terms were related to light sensing and harvesting, cold acclimation and growth speed, such as photosynthesis, light harvesting in photosystem I (GO:0009768), auxin biosynthesis (GO:0009851) and cold acclimation (GO:0009631) (Fig. 5). Therefore, the following analysis of DEGs about reblooming mainly focused on the circadian rhythm related genes, vernalization genes and auxin metabolism related genes.

KEGG is a database for understanding the high-level functions and the biological system, such as cell, organisms and ecosystem, from molecular-level information, especially large-scale molecular datasets generated by genome sequencing and other high-throughput experimental technologies [32]. Among the top 20 enriched KEGG pathways, the “plant hormone signal transduction” under the “environmental information processing” category and the “circadian rhythm-plant” under the “organismal systems” category, were closely related to flowering in plants (Fig. 4). The following DEG analysis and key gene filtering mainly focused on these two pathways.

In the comparison of the floral initiation stages of spring flowering in once-bloomers and rebloomers (OB-T1 vs RB-T1), the numbers of DEGs annotated into these two pathways were the smallest (two DEGs in “plant hormone signal transduction” pathway and five in “circadian rhythm-plant” pathway) (Additional file 2: Fig. S4A). It could be concluded that the gene expression levels of once-bloomers and rebloomers were similar in the spring flower initiation stage. When the floral initiation stage of spring flowering (T1) was compared with the floral initiation stage of autumn flowering (T5), the number of DEGs was much larger (Additional file 2: Fig. S4B-C), indicating the different gene expression patterns in the first and second floral initiation stages.

### The putative regulatory network may provide clues to the regulation of reblooming

The putative regulatory network was based on the comprehensive analysis of the circadian rhythm, the temperature insensitivity and the growth speed. The daily rotation of the Earth alters the environment regularly, the most striking of which is the diurnal change in light and temperature. This periodicity is mirrored by daily periodicity in the behavior and physiology of organisms [15]. In plants, the face-to-sun character of sunflowers is the most common performance of circadian rhythms. The daily biological rhythms are controlled by the circadian clock, an internal timer or oscillator that keeps approximately 24-h time. From a more general perspective, the circadian clock is also important for the seasonal processes, including the seasonal flowering, the transition from vegetative to reproductive growth and the onset of dormancy in plants [33].

In the hub gene analysis, *IgPHYA*, *IgGI* and *IgFT* are among the top 10 nodes with most interactions to other genes, indicating their important roles in the regulation of reblooming (Additional files 2: Fig. S7). In the circadian rhythm regulatory network, the photoreceptors *PHYA* (a far-red-light receptor) and *CRY2* (a blue-light receptor) could stabilize the expression of *CONSTANS* (*CO*) at the end of a long day [34, 35]. *PHYA* also inhibits the *COP-SPA1* complex, but the mechanism is unknown yet [36]. As an essential gate connecting the central oscillator with many physiological processes [37], *GI* has been proposed as a hub gene regulating key features of plant life, such as flowering, salt and freezing tolerance [37, 38]. In the putative pathway, the expression level of *PHYA* and *GI* in T5 was much higher than that in T1, which resulted in the different expression of its downstream gene *CO* in OB-T1 vs RB-T5, and the rapid increase of *FT* and *AP1* on the stage of second flower initiation (Fig. 8). This further supported that the circadian rhythm components *PHYA* and *GI* may play the gating role in reblooming.

Besides the regulation of the circadian clock, some growth speed related genes were detected as DEGs and may exert some influence on the formation of rebloomers. *ARF* was differentially expressed in all of the comparisons between two adjacent periods in rebloomers, but its expression was similar in once-bloomers (Fig. 7d). Therefore, *ARF* may play an essential role to accelerate the vegetative growth and to enable the reblooming. The phytohormone auxin mediates a stunning array of plant development through the functions of *ARFs*. The *ARF* transcription factor is an essential regulator of growth and development, and functions as a protein family comprising 10–43 members identified in different plant species [39].

In addition to the circadian rhythm and growth speed, the different temperature sensitivity between once-bloomers and rebloomers was detected. The second floral initiation occurred in early June, and the second blooming occurred in October. Given that there was no vernalization from floral initiation to full bloom, we supposed that the rebloomers were temperature insensitive. *FRI* and *FLC* are important vernalization pathway genes isolated from winter annual Arabidopsis accessions. *FRI* encodes a coiled-coil protein and could increase the RNA levels of *FLC*, a transcriptional repressor in MADS-box protein family [40]. Vernalization accelerates flowering by downregulating the expression of *FLC* and thus antagonizes the effect of *FRI* [40–42]. In the flower development stages, although the expression of *FRI* was enhanced in the second floral initiation (T5), its downstream gene *FLC* was not screened as a DEG (Fig. 8, Additional file 2: Fig. S6). However, another flowering repressor *SVP* was detected as a DEG, whose expression level in RB-T5 was much lower than those in OB-T1 and RB-T1 (Fig. 8), which may promote the second floral initiation. The expression of *TFL1*, an important flowering repressor, was repressed during the floral initiation period in remontant rose and strawberry [11, 12, 24]. However, *TFL1* was highly expressed in the second floral initiation period of reblooming iris, the reason of which need further investigation.

## Conclusions

An F_1_ hybrid population, including once-bloomers and rebloomers, was created for transcriptome sequencing, which provided the first comprehensive floral development transcriptome data about once-blooming and reblooming bearded iris. In our putative flowering regulatory network, the upstream circadian rhythm genes *IgPHYA* and *IgGI* regulated the auxin related gene *IgARF*, promoting the growth in rebloomers and enabling two cycles of flowering annually. The vernalization pathway genes, *IgPIE1*, *IgFRI* and *IgVIN3*, were expressed differentially in rebloomers and once-bloomers, but their target gene *IgFLC* had similar expression profile. All the upstream signals integrated and resulted in the lower expression of *IgSVP* (a flowering repressor) in the second floral initiation stage, thereby causing the reblooming characteristics. These investigations provide the first insight into the potential mechanism controlling the reblooming of bearded iris, and shed some light on the future molecular breeding of more reblooming perennials.

## Methods

### Plant materials

The *I. germanica* cultivars ‘D’ (Accession No. R20130001) and ‘H’ (Accession No. R20130005), introduced from the Hall’s Flower Garden (West Alexandria, Ohio, USA), have been conserved in the scientific base of Beijing Botanical Garden, Beijing, China for many years. The cultivar ‘D’ is bred by hybridizer Earl Hall (West Alexandria, Ohio, USA), and ‘H’ is bred by hybridizer Sterling U. Innerst (Dover, Pennsylvania, USA).

### Construction of sequencing hybrid population

The F_1_ generation of *I. germanica* ‘D’ × *I. germanica* ‘H’ was obtained by artificial pollination. To avoid contamination from other pollen, artificial emasculation (namely removing three falls and stamens and covering the bags), was carried out the day before blooming. The flowers were covered with bags after artificial emasculation. To make negative controls for each hybridization combination, ten maternal individuals were covered with bags from before flowering to after pollination, but no seeds were produced.

The experimental materials were all cultivated in the Changping District of Beijing, China (40°09′15″N, 116°27′44″E). All plant materials were given the same condition of soil, moisture, temperature and light, in order to avoid any influence from the external environment.

### Phenotypical character analysis of hybrids

The individuals in the F_1_ generation were distributed in a randomized complete block design. During the blooming stage, sixty individuals were selected randomly from each population, and ten key ornamental traits were measured, including plant height (PH; the vertical distance from the soil level to the peak of the top leaf), leaf length (LL; the length of the longest leaf), number of leaves per blooming stem (NLBS), number of leaves per non-blooming stem (NLNS), number of stems (NS), number of flowers per scape (NFS), height of the individual flower (HF; the vertical distance between the fall’s lowest part and the standard’s highest part), diameter of flower (DF; the largest parallel distance between two falls), length of fall (LF) and width of fall (WF). All the measurements were carried out in triplicate for each plant.

The significant differences among various characters were tested with one-way ANOVA using Duncan’s new multiple range in the SPSS 22.0 software. The seed setting rate was estimated as followed:

$$ \mathrm{Seed}\ \mathrm{setting}\ \mathrm{rate}\ \left(\%\right)=\frac{\mathrm{Number}\ \mathrm{of}\ \mathrm{flowers}\ \mathrm{with}\ \mathrm{set}\ \mathrm{seeds}}{\mathrm{Number}\ \mathrm{of}\ \mathrm{flowers}\ \mathrm{pollinated}}\times 100\%. $$

The flowering rate was calculated as followed:

$$ \mathrm{Flowering}\ \mathrm{rate}\ \left(\%\right)=\frac{\mathrm{Number}\ \mathrm{of}\ \mathrm{blooming}\ \mathrm{plants}}{\mathrm{Total}\ \mathrm{number}\ \mathrm{of}\ \mathrm{plants}}\times 100\%. $$

### RNA extraction and RNA-Seq

Total RNA was extracted from the leaves, flower buds or shoot meristem in six developmental stages from reblooming and once-blooming F_1_ individuals, respectively (Table 2, Additional file 2: Fig. S1), using the Quick RNA Isolation Kit (Huayueyang, Beijing, China). The quality of RNA was measured by agarose gel electrophoresis, and the RNA purity was measured by a NanoPhotometer spectrophotometer (IMPLEN, CA, USA). The RNA concentration was measured with the Qubit RNA Assay Kit in Qubit 2.0 Fluorometer (Life Technologies, CA, USA). The cDNA library was sequenced using the Illumina HiSeq 2000 high throughput sequencing platform, and a large number of high-quality reads were obtained. Most of the base quality scores reached or exceeded Q30.

### De novo assembly and functional annotation

Before de novo assembly, the raw reads were firstly processed with in-house Perl scripts. Clean reads were obtained by removing reads containing adapters, reads with unknown base “N” (where the “N” ratio was more than 10%) and low quality reads (more than 50% of the bases with a quality score (Q) < 10) from raw data. De novo assembly of the *I. germanica* transcriptome was performed on the clean data using the method of Trinity [43]. The left files (read1 files) from all samples were pooled into one big “left.fq” file, and right files (read2 files) into one big “right.fq” file. Transcriptome assembly was accomplished based on the “left.fq” and “right.fq” using Trinity with min_kmer_cov set to 2 by default.

The reads from each sequencing sample were compared to NCBI Nr, GO, COG, KEGG and Swiss-Prot databases with BLAST software (version 2.2.26).

### DEG functional annotation and enrichment analysis

Differential expression analysis of two groups was performed using the DESeq R package (1.10.1). The resulting *P*-values were adjusted using the Benjamini and Hochberg’s approach [44] for controlling the false discovery rate (FDR). Genes with an adjusted *P*-value less than 0.05 were assigned as differentially expressed.

In the GO enrichment analysis, the DEGs were implemented using topGO R packages based on the Kolmogorov–Smirnov test. In order to further reduce the redundant GO terms, REVIGO analysis [45] was carried out according to semantic similarity and SimRel semantic similarity measure, with an allowed similarity of 0.7 (medium) [46], after which the results were graphically displayed. KOBAS software [47] was applied to test the statistical enrichment of differentially expressed genes in KEGG (http://www.genome.jp/kegg/) pathways.

### Protein-protein interaction (PPI) network analysis

The putative DEGs in the reblooming network were analyzed in the Search Tool for Retrieval of Interacting Genes/Proteins (STRING) online database, in order to construct the PPI network (https://string-db.org/) [48]. Then the credible PPI interactions were further analyzed in the Cytoscape software to visualize the hub genes [49].

### Quantitative real-time PCR (qRT-PCR) validation

Ten DEGs were selected randomly for qRT-PCR. The primers of all the selected DEGs were designed by Primer Premier 5 (Additional file 1: Table S11). A 25 μL reaction mixture was added into 96-well plates, including 12.5 μL 2 × SYBR Green Master, 1 μL forward primer (20 μM), 1 μL reverse primer (20 μM), 2 μL cDNA and 8.5 μL ddH_2_O. Reactions were performed with Bio-Rad CFX96 Real-Time PCR Detection System: pre-incubation (95 °C for 30 s); two-step amplification (95 °C for 5 s; 59.5 °C for 30 s; 40 cycles); melting (95 °C for 10 s; 65 °C to 95 °C, increment 0.5 °C, for 5 s). All the experiments were carried out with three biological replicates, and each biological replicate had three technical replicates. The relative expression level was calculated according to the 2^-ΔΔCt^ algorithm, and was normalized to *ACTIN* gene with stable differential expression in this work.

## Supplementary information

**Additional file 1: Table S1.** The seed setting rates and flowering rates of F_1_ in spring and autumn. **Table S2.** Summary of Illumina transcriptome assembly. **Table S3.** The functional annotations of unigenes against the public databases. **Table S4.** The three GO functional categories of all the unigenes. **Table S5.** The annotations of unigenes by KEGG pathway analysis.**Table S6.** The expression levels and annotations of the DEGs. **Table S7.** The significantly enriched KEGG pathways of DEGs in OB-T1 vs RB-T1. The pathways were presented according to the number of DEGs in this pathway. **Table S8.** The significantly enriched KEGG pathways in DEGs in RB-T1 vs RB-T5. The pathways were presented according to the number of DEGs in this pathway. **Table S9.** The significantly enriched KEGG pathways in DEGs in OB-T1 vs RB-T5. The pathways were presented according to the number of DEGs in this pathway. **Table S10.** The GO enrichment analysis of DEGs. **Table S11.** Primers used in qRT-PCR.

**Additional file 2: Fig. S1.** The apical meristem and the appearances of *I. germanica* in the six sampled stages. (A) The floral initiation stage of spring flowering (T1) and autumn flowering (T5). (B) The stage after entering dormancy (T2). (C) The stage after dormancy release (T3). (D) The bud stage of spring flowering (T4) and autumn flowering (T6). **Fig. S2.** The length distribution of the assembled unigenes. **Fig. S3.** Volcano plots of DEGs in *I. germanica* transcriptome OB-T1 vs RB-T1 (A), RB-T1 vs RB-T5 (B), OB-T1 vs RB-T5 (C). Each dot represents a gene. Black dots represent the unchanged unigenes. Green dots represent the down-regulated DEGs and red dots represent the up-regulated DEGs. **Fig. S4.** KEGG pathways significantly enriched in DEGs in the comparisons of OB-T1 vs RB-T1 (A), RB-T1 vs RB-T5 (B) and OB-T1 vs RB-T5 (C). **Fig. S5.** GO terms significantly enriched in DEGs in the comparisons of OB-T1 vs RB-T1 (A), RB-T1 vs RB-T5 (B) and OB-T1 vs RB-T5 (C). **Fig. S6.** The heat map of DEGs in vernalization pathway. Dark-blue indicates a relative increase of expression, and light-blue represents a relative decrease of expression. *PHOTOPERIOD-INDEPENDENT EARLY FLOWERING 1* (*PIE1*); *FRIGIDA* (*FRI*); *VERNALIZATION INSENSITIVE 3* (*VIN3*); *FLOWERING LOCUS C* (*FLC*). **Fig. S7.** Protein-protein interaction networks of the putative reblooming-regulatory DEGs identified by RNA-seq. (A) Protein-protein interactions among reblooming-regulatory DEGs in Fig. 8. Nodes represent proteins while edges stand for the predicted functional interactions. (B) The graphical depiction of the top 10 interaction nodes in the hub module. Different color represents different hub gene analysis rank.

## Data Availability

The raw reads have been submitted to NCBI SRA (Sequence Read Archive, http://www.ncbi.nlm.nih.gov/sra/), under the accession number PRJNA638517, and NCBI TSA (Transcriptome Shotgun Assembly, https://www.ncbi.nlm.nih.gov/nucleotide/), under the accession number GIQU00000000.

## Abbreviations

*AP1*: *APETALA 1*
*ARF*: *AUXIN RESPONSE FACTOR*
*CCA1*: *CIRCADIAN CLOCK ASSOCIATED 1*
*CO*: *CONSTANS*
COG: Clusters of orthologous groups
*CRY*: *CRYPTOCHROME*
DEG: Differentially expressed gene
DF: Diameter of flower
ED: Extended-day
*EXPA16*: *EXPANSIN-A16-LIKE*
FDR: False discovery rate
*FLC*: *FLOWERING LOCUS C*
*FRI*: *FRIGIDA*
*FT*: *FLOWERING LOCUS T*
*GI*: *GIGANTEA*
GO: Gene ontology
HF: Height of individual flower
KEGG: Kyoto encyclopedia of genes and genomes
LF: Length of fall
LL: Leaf length
NFS: Number of flowers per scape
NLBS: Number of leaves per blooming stem
NLNS: Number of leaves per non-blooming stem
Nr: Non-redundant protein sequences
NS: Number of stems
OB: Once-bloomers
OB-T1: The floral initiation stage of spring flowering in once-bloomers
OB-T2: The stage after entering dormancy in once-bloomers
PH: Plant height
*PHYA*: *PHYTOCHROME A*
*PIE1*: *PHOTOPERIOD-INDEPENDENT EARLY FLOWERING 1*
PPI: Protein-protein interaction
*PRR9*: *PSEUDO-RESPONSE REGULATOR 9*
PV_s-a_: Difference between phenotypic value in spring and autumn
RB: Rebloomers
RB-T1: The floral initiation stage of spring flowering in rebloomers
RB-T2: The stage after entering dormancy in rebloomers
RB-T5: The floral initiation stage of autumn flowering in rebloomers
REVIGO: Reduce and Visualize GO
SD: Short-day
*SPA1*: *SUPPRESSOR OF PHYTOCHROME A1*
*SVP*: *SHORT VEGETATIVE PERIOD*
T1: The floral initiation stage of spring flowering
T4: The bud stage of spring flowering
T5: The floral initiation stage of autumn flowering
*TBL*: *TRICHOME BRIRFRINGENCE-LIKE*
*TFL1*: *TERMINAL FLOWER 1*
*VIN3*: *VERNALIZATION INSENSITIVE 3*
WF: Width of fall
