# Supplementary material for: To bloom once or more times: the reblooming mechanisms of Iris germanica revealed by transcriptome profiling
Source: BMC Genomics. 2020 Aug 12;21:553. doi: 10.1186/s12864-020-06951-x (PMC7430825; doi:10.1186/s12864-020-06951-x)
Supplement: Supplementary file 2 — Additional file 2: Fig. S1. The apical meristem and the appearances of I. germanica in the six sampled stages. (A) The floral initiation stage of spring flowering (T1) and autumn flowering (T5). (B) The stage after entering dormancy (T2). (C) The stage after dormancy release (T3). (D) The bud stage of spring flowering (T4) and autumn flowering (T6). Fig. S2. The length distribution of the assembled unigenes. Fig. S3. Volcano plots of DEGs in I. germanica transcriptome OB-T1 vs RB-T1 (A), RB-T1 vs RB-T5 (B), OB-T1 vs RB-T5 (C). Each dot represents a gene. Black dots represent the unchanged unigenes. Green dots represent the down-regulated DEGs and red dots represent the up-regulated DEGs. Fig. S4. KEGG pathways significantly enriched in DEGs in the comparisons of OB-T1 vs RB-T1 (A), RB-T1 vs RB-T5 (B) and OB-T1 vs RB-T5 (C). Fig. S5. GO terms significantly enriched in DEGs in the comparisons of OB-T1 vs RB-T1 (A), RB-T1 vs RB-T5 (B) and OB-T1 vs RB-T5 (C). Fig. S6. The heat map of DEGs in vernalization pathway. Dark-blue indicates a relative increase of expression, and light-blue represents a relative decrease of expression. PHOTOPERIOD-INDEPENDENT EARLY FLOWERING 1 (PIE1); FRIGIDA (FRI); VERNALIZATION INSENSITIVE 3 (VIN3); FLOWERING LOCUS C (FLC). Fig. S7. Protein-protein interaction networks of the putative reblooming-regulatory DEGs identified by RNA-seq. (A) Protein-protein interactions among reblooming-regulatory DEGs in Fig. 8. Nodes represent proteins while edges stand for the predicted functional interactions. (B) The graphical depiction of the top 10 interaction nodes in the hub module. Different color represents different hub gene analysis rank. [file 12864_2020_6951_MOESM2_ESM.docx]

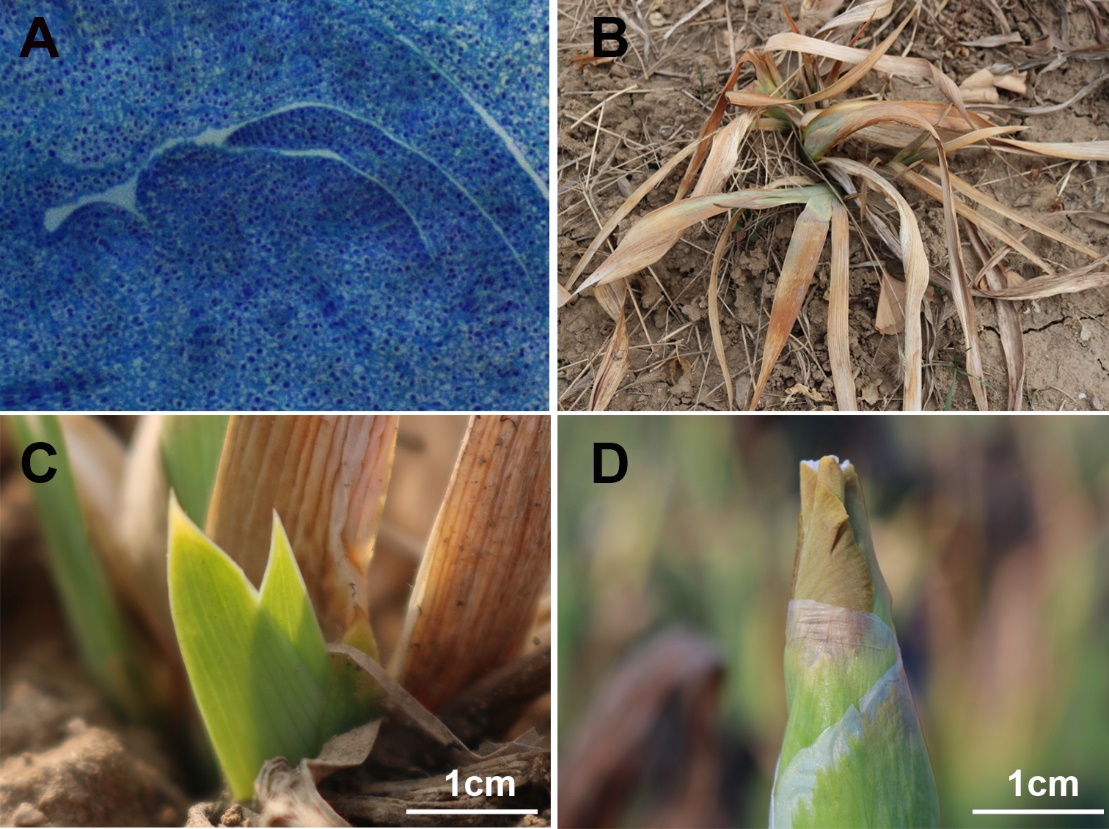


**Additional file 2: Figure S1**


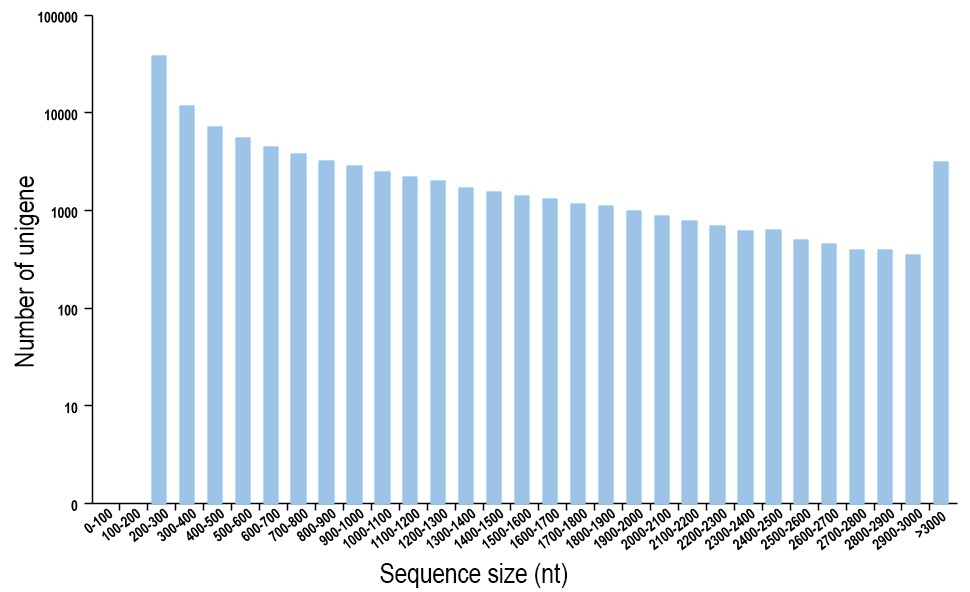


**Additional file 2: Figure S2**


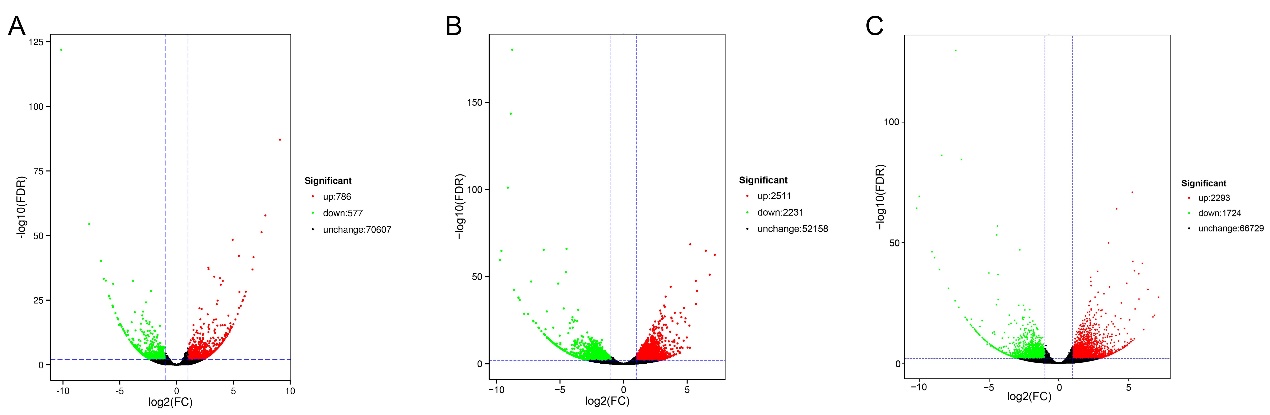


**Additional file 2: Figure S3**

**
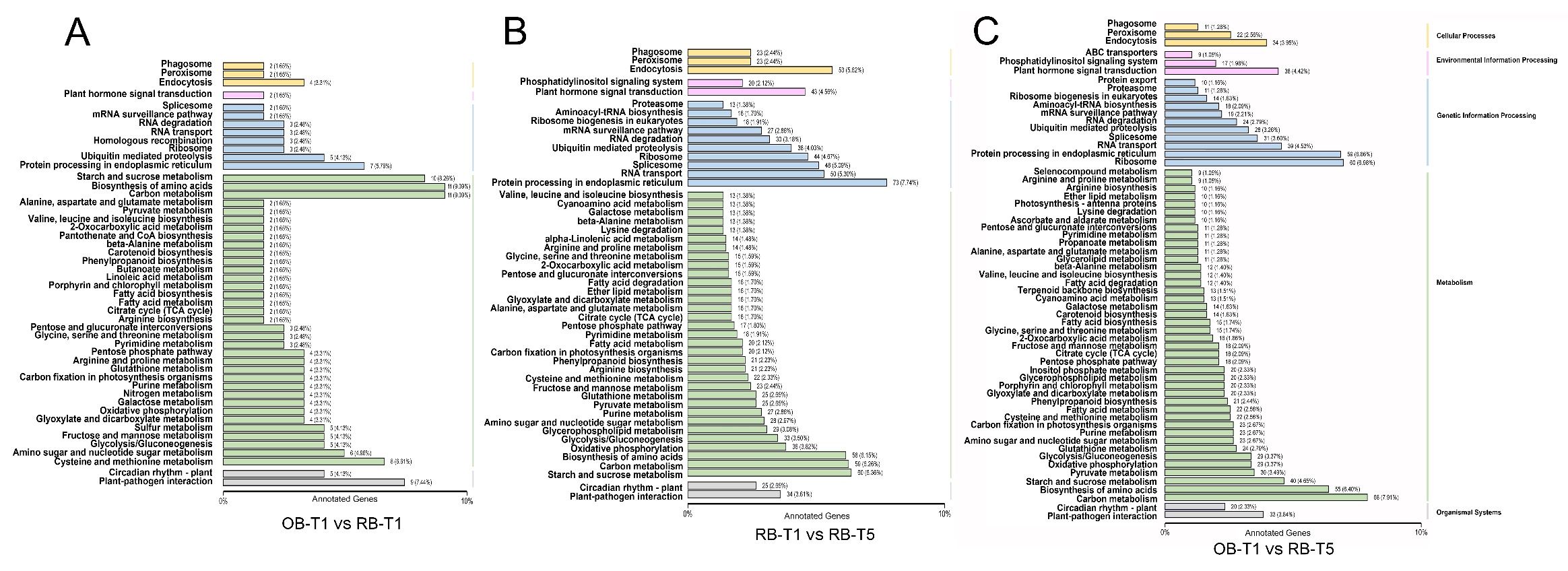
**

**Additional file 2: Figure S4**

**
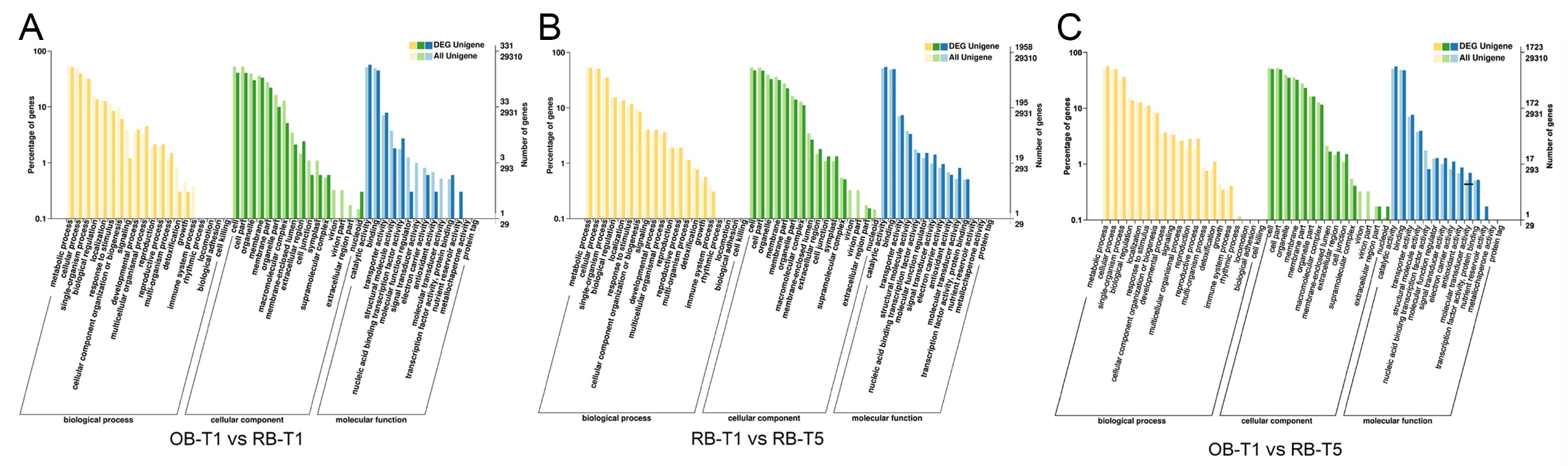
**

**Additional file 2: Figure S5**


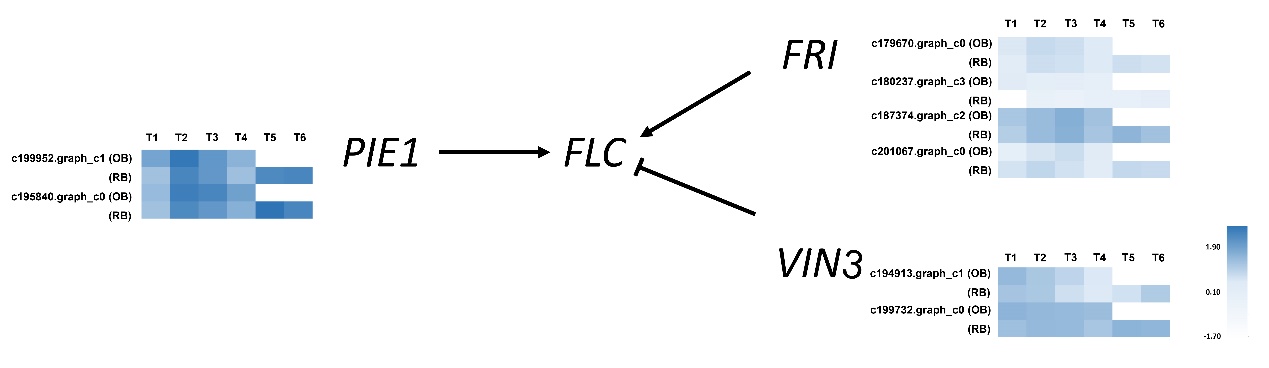


**Additional file 2: Figure S6**

**
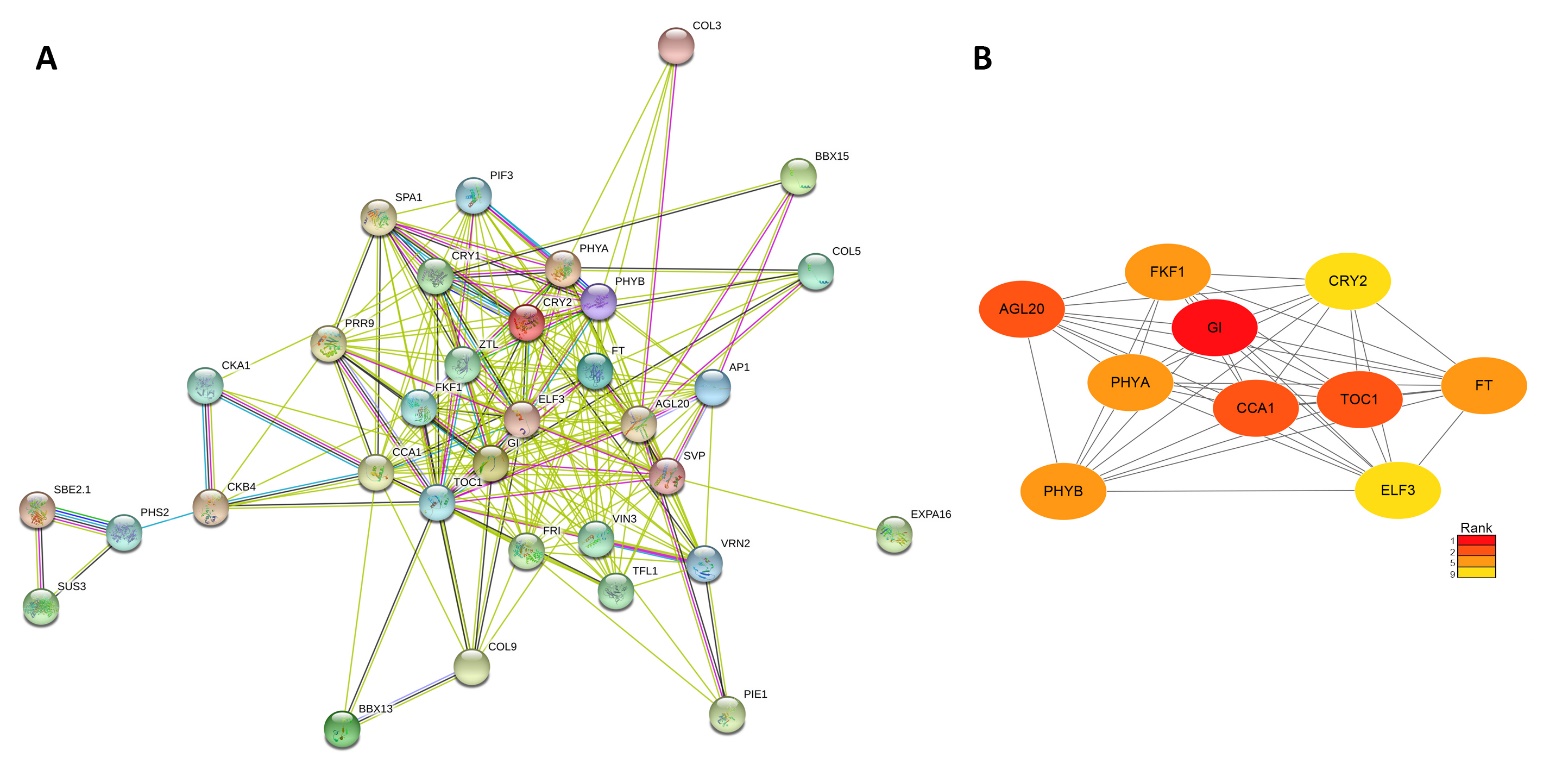
**

**Additional file 2: Figure S7**
